# Association between alkaline phosphatase to albumin ratio and mortality among patients with sepsis

**DOI:** 10.1038/s41598-024-53384-7

**Published:** 2024-02-07

**Authors:** Shuyue Liu, Kai Zhao, Chunhong Shao, Lulu Xu, Xianglun Cui, Yong Wang

**Affiliations:** 1 Department of Laboratory Medicine, Shandong Provincial Hospital Affiliated to Shandong First Medical University, Jinan, 250021 Shandong China; 2 Information Network Management Office, Shandong Provincial Hospital Affiliated to Shandong First Medical University, Jinan, 250021 Shandong China; 3 Department of Clinical Laboratory, Shandong Provincial Hospital Affiliated to Shandong First Medical University, No. 324, Jingwu Weiqi Road, Huaiyin District, Jinan, 250021 Shandong China

**Keywords:** Biomarkers, Risk factors

## Abstract

The alkaline phosphatase-to-albumin ratio (APAR) is correlated to worse prognosis in coronary artery disease, cancer, and acute renal failure. However, the relationship between APAR and sepsis prognosis has received little research. The content of this research was to investigate the prognostic relationship between APAR and sepsis. And validate the stability of the correlation in 90-days and 1-year mortality. Retrospective cohort research was conducted basing MIMIC-IV database (version 2.0). The hazard ratio (HR) and 95% confidence interval (Cl) were computed using multivariate Cox regression analysis. In addition, plots of survival curves and subgroup analyzes were conducted. Receiver operating characteristic (ROC) curves were also used. 9741 participants were included in this investigation. The 90-days mortality was 32.8%, and the 1-year mortality was 42.0%. After controlling for confounders, the adjusted HRs (95% CI) for tertile 2 (2.2–3.8) and tertile 3 (> 3.8) were 1.37 (1.25–1.51) and 1.74 (1.58–1.91), respectively. The Kaplan–Meier curve analysis showed a higher probability of 90-days death in the higher APAR group. The area under the curve (AUC) of APAR was 0.674 and could reach 0.709 after combining the Oxford Acute Severity of Illness Score (OASIS). This study demonstrates that APAR is significantly related to bad clinical outcomes in sepsis.

## Introduction

Sepsis, a severe global public health problem, is an organ dysfunction due to infection-induced immune dysregulation^1^. Some progress has been made in recognizing and managing clinical sepsis, but morbidity and mortality remain high. A recent study showed that death caused by sepsis accounted for 19.7% of fatalities worldwide^2^. Therefore, effective identification of high-risk patients early is crucial, as it has the potential to reduce the rate of multiorgan failure and mortality.

It has been demonstrated that some scoring methods are correlated with the prognosis of sepsis patients^3–5^. However, they are not readily available due to the large number of indicators involved in some complex scales, such as the Oxford Acute Severity of Illness Score (OASIS). Conducting dynamic monitoring is burdensome for both patients and physicians. Furthermore, the Sequential Organ Failure Assessment (SOFA) score has limitations in assessing the mid- and long-term prognosis of patients with severe sepsis. Therefore, it is necessary to find biomarkers with good predictive value and economic convenience to assist doctors in recognizing high-risk patients.

Albumin is a common and multifunctional protein that predicts prognosis in various diseases, such as infections, acute renal failure, and acute coronary syndromes^6–8^. Studies have established that hypoalbuminemia is associated with infection-related death^9,10^. Alkaline phosphatase has also been investigated for its prognostic value in patients with bacteremia^11^. Recent investigations have revealed that the alkaline phosphatase-to-albumin ratio (APAR) is an essential biomarker of prognosis in several diseases, including cancer, coronary heart disease, and acute renal failure^12–14^. In addition, recent studies have designed economical and rapid methods to test APAR^15^. However, there is less research involving the relationship between APAR and adverse outcomes in sepsis.

Based on the investigations above, we speculate that APAR is associated with a prognosis of sepsis. Our article aims to validate whether a simple, low-cost indicator is associated with sepsis death and has good prognostic value. In this article, we will use the MIMIC public database to verify the prognostic value of the APAR indicator for sepsis. This will help doctors determine the prognosis of sepsis more easily and effectively.

## Material and methods

### Database

Data for this analysis were derived from the MIMIC-IV database (version 2.0) on the PhysioNet website, which provides comprehensive data on various intensive care unit (ICU) admissions from 2008 to 2019 at Beth Israel Deaconess Medical Center (BIDMC)^16^. The database contains critical care data on more than 40,000 patients. Users can access each patient’s demographic characteristics, vital signs, laboratory test results, and imaging by using the de-identified number given during admission. The database has played an integral role in advancing a large number of studies in clinical informatics, epidemiology, and machine learning. The first author, Shuyue Liu, is a credentialed user (author certification number: 11515326) who completed the course “Data or Specimens Only Research”. The collection of patient information was reviewed by the Institutional Review Board at the BIDMC.

### Participants

The study population for this study was derived from patients admitted to ICU in the MIMICIV (version 2.0) database between 2008 and 2019, in which we developed criteria to select the target population that met the purpose of our study. The following were inclusion criteria: (1) age ≥ 18 years^17–19^; (2) diagnosis of sepsis within 24 h of admission; (3) we only included data for the first admission to the ICU for patients with multiple admissions to the ICU. Exclusion criteria were as follows: (1) patients without records of hospital time; (2) patients who died or were discharged within 24 h of admission; (3) within 24 h of ICU admission, patients without albumin or alkaline phosphatase levels. Sepsis is clinically defined as a SOFA score ≥ 2 points plus suspected or documented infection^1^. In this study, we defined the suspected infection and calculated the SOFA score by referring to the official open codes given by MIMIC. The definition of suspected infection is sending a pathogenetic test within 24 h after applying antibiotics or applying an antibiotic within 72 h after sending a pathogenetic test^20^. The definition of septic shock is derived from the International Classification of Diseases (ICD) code R6521 (10th revision) and 78,552 (9th revision)^21^.

### Data extraction

We extracted the following variables: Basic demographic information: age, gender, ethnicity, and weight; Vital signs: mean blood pressure (MBP), heart rate, respiratory rate, temperature, and SPO_2_ at ICU admission. The severity of admission: assessed by SOFA score, OASIS, and Charlson comorbidity index (CCI); Laboratory results: white blood cell (WBC), hemoglobin, platelet, glucose, anion gap, chloride, potassium, sodium, albumin, blood urea nitrogen (BUN), creatinine, aspartate aminotransferase (AST), alanine aminotransferase (ALT), alkaline phosphatase (ALP), C-reactive protein (CRP), lactate, and pH (however, the CRP, lactate, and pH variables contained too many missing values to be ultimately included in these covariates). APAR is equal to ALP divided by albumin. Comorbidity disease: myocardial infarction (MI), chronic pulmonary disease (CLD), congestive heart failure (CHF), renal disease, liver disease, severe liver disease, diabetes, and malignant cancer were estimated by the CCI. Infection site: bloodstream infection, genitourinary infection, respiratory infection, gastrointestinal infection, other site, and unknown. Intervention: use of ventilator, renal replacement therapy (RRT), and vasopressor during the first 24 h of ICU admission.

We selected the first set of parameters if the included variables were measured more than once in 24 h^18,19^. In addition, we excluded covariates with missing values ≥ 30% but maintained the study population constant^17,22^.

The visualization of the MIMIC database is made by running Structured Query Language (SQL) using the PgAdmin4 (version 6.15) platform. The open code for extracting data is from the Alistair Johnson homepage on the GitHub website^20^. Furthermore, the extracted data were filtered and combined using STATA (version 17.0) software.

### Outcome

In this study, 90-days mortality was the primary outcome. Secondary outcomes included 1-year mortality and length of stay (LOS) hospital. The date of admission was used as the beginning of the follow-up, and the date of death was obtained from the Massachusetts State Registry of Vital Records and Statistics.

### Statistical analysis

Descriptive statistics: Data for normal continuous variables were expressed as mean ± standard deviation (SD) and compared between groups using one-way analysis. In contrast, skewed distribution data were expressed as median (25th–75th percentile) and compared using the Kruskal–Wallis test. Categorical variables were expressed as frequencies and percentages and analyzed using the Chi-square.

Association between APAR and 90-days mortality: To begin preliminarily exploring the connection between APAR and 90-days mortality, we performed univariate Cox regression analyzes. Confounders were selected based on the *P* values of univariate Cox regression < 0.1 or changes in the effect estimate of more than 10%^23^. Then, multivariate Cox proportional hazards regression analysis between APAR and 90-days mortality was performed. In model 1, the adjustment covariates include age and gender. In model 2, the adjustment covariates include age, gender, OASIS, SOFA score, and CCI. In model 3, the covariates include age, gender, OASIS, SOFA score, CCI, ethnicity, weight, MBP, respiratory rate, heart rate, SpO_2_, temperature, WBC, hemoglobin, platelet, anion gap, potassium, chloride, creatinine, BUN, ALT, AST, MI, CHF, CLD, liver disease, severe liver disease, renal disease, malignant cancer, ventilator use, vasopressor use, and RRT use. We categorized the study population into three equal parts based on the sample size and the value of APAR. The tertile of APAR divided the participants into three groups (T1: ≤ 2.2; T2: 2.2–3.8; T3: > 3.8). We also converted APAR into categorical variables according to tertile and calculated *P* values for the trend.

Cumulative hazard at different APAR levels: Compared the cumulative hazard for different levels of APAR using Kaplan–Meier survival analysis and the log-rank test.

Stratified and interaction analysis: Interaction and stratification analysis was applied to examine whether the associations varied for subgroups, including age, gender, septic shock, MI, CHF, CLD, renal disease, liver disease, severe liver disease, malignant cancer, and diabetes.

Receiver operating characteristic (ROC) curve analysis: ROC curves and area under the curve (AUC) were used to compare APAR with OASIS and SOFA scores to further evaluate APAR’s prediction power.

Sensitivity analysis: We performed three sensitivity analyzes. First, we repeated the analysis using 1-year mortality as the outcome. Then, after patients with missing values were eliminated, sensitivity analysis was performed. Furthermore, we also consider the infusion of human serum albumin. Therefore, after excluding patients who received human serum albumin transfusions 48 h before the test of albumin, another sensitivity analysis was conducted.

After removing variables with more than 30% missing, the proportion of missing data for the remaining variables was less than 5%. The missing values were imputed with the median of non-missing values. Outlier expression is defined as values that greater than the 99th percentile or lower than the 1st percentile. Data with outliers are assigned to the 99th percentile and the 1st percentile, respectively.

All statistical analyzes were conducted using EmpowerStats (www. empowerstats.com, X&Y solutions, Inc. Boston MA) and R software version 3.6.1 (http://www.r-project.org). A two-sided *P* < 0.05 was considered statistically significant.

### Sample size calculation

Based on a retrospective study by Xia et al. in the MIMIC III database, the AUC of the APAR and SOFA ratios were 0.616 and 0.558, respectively^14^. We used this as a reference to select a power of 90% and a significance level of 0.05, with a minimum sample size of 1184 patients, to test APAR’s prognostic value. Patients eligible for inclusion in this database were 9741 patients. Therefore, we included all for analysis to increase the power of the study further.

### Ethical approval and informed consent

The Institutional Review Boards at the BIDMC and Massachusetts Institute of Technology (MIT) have reviewed the studies, which have been approved for a waiver of informed consent. The research was conducted in accordance with the Declaration of Helsinki.

## Results

### Population

**Figure 1:**
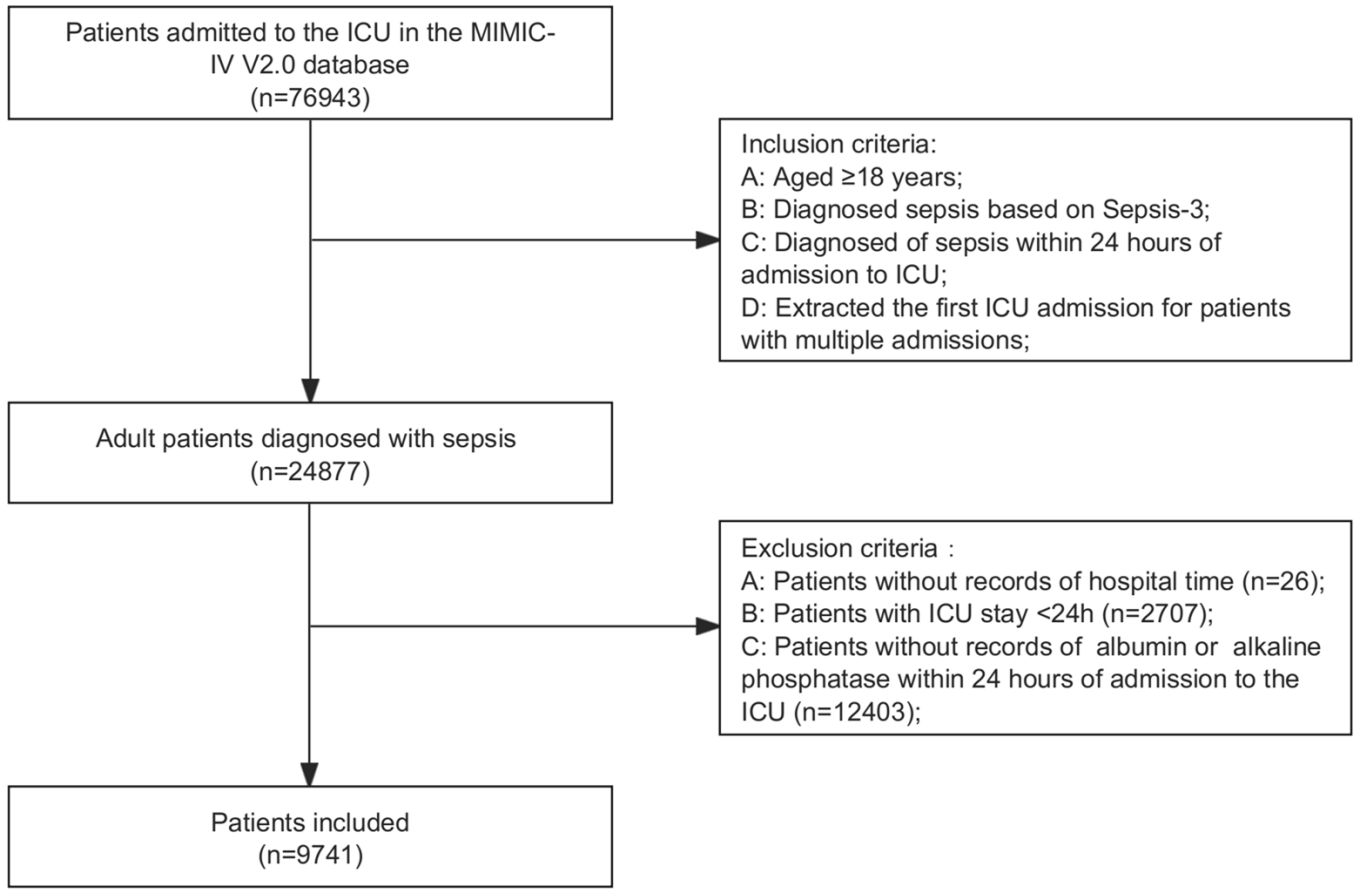
Flowchart of study patients.

The MIMIC-IV database (version 2.0) contains records on 76,943 ICU admissions (Fig. 1). There are 24,877 patients who met the inclusion requirements. After excluding 2707 patients due to ICU stay < 24 h, and 12,403 patients without APAR value or in-hospital time, the analysis comprised 9741 patients.

### Baseline characteristics

Table 1 shows the participant baseline characteristics. Overall, the average age of the patients was 65.2 years. 63.9% of patients were white, and 43.0% of them were female. The whole cohort also was described hierarchically based on APAR tertile. Patients in the highest tertile of APAR had higher OASIS, SOFA scores, CCI, length of hospital stays, 90-days mortality, and 1-year mortality. The most common sites of infection were the bloodstream and genitourinary systems. In addition, the site of infection was unknown in 20.4% of sepsis patients. In addition, patients with higher APAR have a higher probability of combined liver disease, severe liver disease, diabetes, renal disease, malignant cancer, and septic shock.

**Table 1:** Baseline characteristics of participants.

Variables	All patients	T1	T2	T3	*P*-value
		APAR ≤ 2.2	2.2 < APAR ≤ 3.8	APAR > 3.8	
	(n = 9741)	(n = 3290)	(n = 3193)	(n = 3258)	
Age (years)	65.2 ± 16.7	64.4 ± 17.4	65.7 ± 16.6	65.4 ± 15.9	0.002
Gender, female, n (%)	4190 (43.0)	1301 (39.5)	1402 (43.9)	1487 (45.6)	< 0.001
Ethnicity, white, n (%)	6221 (63.9)	2069 (62.9)	2053 (64.3)	2099 (64.4)	0.356
Weight (kg)	78.5 (66.0, 94.5)	79.4 (67.0, 95.1)	79.0 (66.5, 95.4)	76.7 (64.4, 92.2)	< 0.001
Vital signs					
MBP (mmHg)	82.1 ± 18.6	84.3 ± 18.5	82.6 ± 18.9	79.6 ± 18.1	< 0.001
Heart rate (bmp)	93.9 ± 20.9	91.4 ± 20.4	94.2 ± 20.9	96.3 ± 21.0	< 0.001
Respiratory rate (bmp)	20.0 (16.0, 24.0)	19.0 (16.0, 23.0)	20.0 (16.0, 24.0)	20.0 (17.0, 25.0)	< 0.001
Temperature (℃)	36.8 ± 0.9	36.8 ± 0.9	36.8 ± 0.9	36.7 ± 0.9	< 0.001
SpO_2_ (%)	98.0 (95.0, 100.0)	98.0 (95.0, 100.0)	98.0 (95.0, 100.0)	97.0 (95.0, 100.0)	< 0.001
Scoring systems					
OASIS	36.7 ± 9.5	35.9 ± 9.2	37.1 ± 9.4	37.1 ± 10.0	< 0.001
SOFA score	3.0 (2.0, 5.0)	3.0 (2.0, 4.0)	3.0 (2.0, 5.0)	4.0 (2.0, 5.0)	< 0.001
Charlson comorbidity index	6.1 ± 3.0	5.4 ± 2.9	6.2 ± 3.0	6.8 ± 3.1	< 0.001
Laboratory results					
WBC (k/uL)	11.8 (7.8, 17.1)	10.9 (7.5, 15.5)	11.8 (7.8, 17.2)	12.9 (8.0, 18.9)	< 0.001
Hemoglobin (g/dL)	10.9 ± 2.5	11.6 ± 2.6	11.0 ± 2.5	10.2 ± 2.2	< 0.001
Platelet (k/uL)	190.0 (124.0, 271.0)	192.0 (136.2, 256.0)	194.0 (126.0, 279.0)	182.0 (108.0, 284.8)	0.002
Glucose (mg/dL)	133.0 (106.0, 179.0)	137.0 (110.0, 181.0)	138.0 (108.0, 188.0)	126.5 (99.0, 172.0)	< 0.001
Anion gap (mEq/L)	16.0 (13.0, 20.0)	16.0 (13.0, 19.0)	16.0 (13.0, 20.0)	17.0 (14.0, 20.0)	< 0.001
Potassium (mEq/L)	4.4 ± 1.0	4.3 ± 1.0	4.4 ± 1.0	4.4 ± 1.0	0.006
Chloride (mEq/L)	102.1 ± 7.4	103.1 ± 6.9	102.1 ± 7.5	101.1 ± 7.8	< 0.001
Sodium (mEq/L)	137.6 ± 6.1	138.4 ± 5.5	137.9 ± 6.0	136.4 ± 6.6	< 0.001
Albumin (g/L)	31.3 ± 6.9	34.7 ± 6.3	31.6 ± 6.3	27.6 ± 6.2	< 0.001
Creatinine (mg/dL)	1.2 (0.8, 2.0)	1.1 (0.8, 1.7)	1.3 (0.8, 2.2)	1.3 (0.8, 2.2)	< 0.001
BUN (mg/dL)	25.0 (16.0, 43.0)	22.0 (15.0, 36.0)	26.0 (16.0, 44.0)	29.0 (17.0, 48.0)	< 0.001
ALT (IU/L)	30.0 (17.0, 70.0)	24.0 (15.0, 45.8)	28.0 (17.0, 59.0)	45.0 (23.0, 116.0)	< 0.001
AST (IU/L)	46.0 (26.0, 114.0)	36.0 (22.2, 72.0)	42.0 (24.0, 96.0)	71.0 (36.0, 177.8)	< 0.001
ALP (IU/L)	89.0 (63.0, 136.0)	57.0 (46.0, 67.0)	89.0 (76.0, 104.0)	171.0 (132.0, 254.0)	< 0.001
APAR	2.8 (1.9, 4.7)	1.7 (1.4, 1.9)	2.8 (2.5, 3.3)	6.1 (4.7, 9.4)	< 0.001
Comorbidity disease					
Septic shock, n (%)	2551 (26.2)	562 (17.1)	806 (25.2)	1183 (36.3)	< 0.001
Myocardial infarction, n (%)	1699 (17.4)	604 (18.4)	598 (18.7)	497 (15.3)	< 0.001
Congestive heart failure, n (%)	2934 (30.1)	888 (27.0)	1075 (33.7)	971 (29.8)	< 0.001
Chronic pulmonary disease, n (%)	2529 (26.0)	831 (25.3)	896 (28.1)	802 (24.6)	0.004
Liver disease, n (%)	2517 (25.8)	562 (17.1)	809 (25.3)	1146 (35.2)	< 0.001
Severe liver disease, n (%)	1310 (13.4)	245 (7.45)	430 (13.47)	635(19.49)	< 0.001
Diabetes, n (%)	3022 (31.0)	904 (27.5)	1028 (32.2)	1090 (33.5)	< 0.001
Renal disease, n (%)	2277 (23.4)	643 (19.5)	797 (25.0)	837 (25.7)	< 0.001
Malignant cancer, n (%)	1558 (16.0)	356 (10.8)	480 (15.0)	722 (22.2)	< 0.001
Infection site					< 0.001
Bloodstream infection, n (%)	1731 (17.8)	686 (20.85)	584 (18.29)	461 (14.15)	
Genitourinary infection, n (%)	5659 (58.1)	1593 (48.42)	1891 (59.22)	2175 (66.76)	
Respiratory infection, n (%)	166 (1.7)	45 (1.37)	64 (2.00)	57 (1.75)	
Gastrointestinal infection, n (%)	131 (1.3)	28 (0.85)	42 (1.32)	61 (1.87)	
Other, n (%)	71 (0.7)	34 (1.03)	24 (0.75)	13 (0.40)	
Unknown, n (%)	1983 (20.4)	904 (27.48)	588 (18.42)	491 (15.07)	
Interventions					
RRT use, n (%)	500 (5.1)	117 (3.6)	170 (5.3)	213 (6.5)	< 0.001
Ventilator use, n (%)	8150 (83.7)	2819 (85.7)	2723 (85.3)	2608 (80.0)	< 0.001
Vasopressor use, n (%)	4527 (46.5)	1464 (44.5)	1498 (46.9)	1565 (48.0)	0.014
Outcome					
LOS hospital (day)	9.3 (5.5, 16.9)	8.9 (5.4, 15.8)	9.5 (5.6, 16.9)	9.7 (5.4, 18.0)	0.013
90-days mortality, n (%)	3194 (32.8)	702 (21.3)	1052 (32.9)	1440 (44.2)	< 0.001
1-year mortality, n (%)	4093 (42.0)	938 (28.5)	1362 (42.7)	1793 (55.0)	< 0.001

MBP, mean blood pressure; OASIS, Oxford Acute Severity of Illness Score; SOFA, Sequential Organ Failure Assessment; WBC, white blood cell; BUN, blood urea nitrogen; ALT, alanine aminotransferase; AST, aspartate aminotransferase; ALP, alkaline phosphatase; APAR, alkaline phosphatase to albumin ratio; RRT, renal replacement therapy; LOS, length of stay.

### Association between APAR and 90-mortality

To investigate potential correlates of 90-days mortality in sepsis, we performed univariate Cox regression analyzes (Supplementary Table 1). In the multivariate Cox regression analysis, changes in effective estimates exceeding 10% or* P* < 0.1 in univariate analyzes will be included as confounders in this study.

Three models were built in this study to explore the association of APAR with 90-days mortality (Table 2). In model 1 (adjusted covariates of age and gender), the effect size for APAR on 90-days mortality increased by one unit and by 6%, and the hazard ratio (HR) and 95% confidence interval (Cl) were 1.06 (1.05–1.06). In model 2 (adjusted covariates of age, gender, OASIS, CCI, and SOFA score), the effect size increased by 5% for each one-unit increase in APAR (HR: 1.05, 95% CI 1.04–1.05). In model 3 (adjusted covariates of age, gender, OASIS, CCI, SOFA score, ethnicity, weight, MBP, respiratory rate, HR, SpO_2_, temperature, WBC, hemoglobin, platelet, potassium, chloride, anion gap, creatinine, BUN, ALT, AST, MI, CLD, CHF, renal disease, liver disease, severe liver disease, malignant cancer, RRT use, and vasopressor use, ventilator use), the effect size increase by 4% (HR: 1.04, 95% CI 1.03–1.04) for every 1 unit increase in APAR. For further analysis, we also converted the continuous variable APAR to a categorical variable. The first tertile of APAR (T1) was employed as the reference. In model 3, even after adjustment for all potential confounders, the adjusted HRs (95% CI) for tertile 3 were 1.74 (1.58–1.91). Moreover, *P* values for trends in different models were statistically significant (all *P* < 0.001).

**Table 2:** Multivariable Cox regression analysis to assess the association between APAR and 90-days mortality.

Variables	Unadjusted model		Model I		Model II		Model III	
	HR (95% CI)	*P*-value	HR (95% CI)	*P*-value	HR (95% CI)	*P*-value	HR (95% CI)	*P*-value
APAR	1.06 (1.05–1.06)	< 0.001	1.06 (1.05–1.06)	< 0.001	1.05 (1.04–1.05)	< 0.001	1.04 (1.03–1.04)	< 0.001
Tertile								
T1 (APAR ≤ 2.2)	Ref		Ref		Ref		Ref	
T2 (2.2 < APAR ≤ 3.8)	1.65 (1.50–1.82)	< 0.001	1.62 (1.47–1.78)	< 0.001	1.44 (1.31–1.59)	< 0.001	1.37 (1.25–1.51)	< 0.001
T3 (APAR > 3.8)	2.40 (2.19–2.62)	< 0.001	2.38 (2.17–2.60)	< 0.001	2.01 (1.83–2.20)	< 0.001	1.74 (1.58–1.91)	< 0.001
*P* for trend		< 0.001		< 0.001		< 0.001		< 0.001

Model I adjusted for age, gender; Model II adjusted for model I plus OASIS, SOFA score, Charlson comorbidity index; Model III adjusted for Model II plus ethnicity, weight, MBP, heart rate, respiratory rate, temperature, SpO_2_, WBC, hemoglobin, platelet, anion gap, potassium, chloride, creatinine, BUN, ALT, AST, myocardial infarction, congestive heart failure, chronic pulmonary disease, liver disease, severe liver disease, renal disease, malignant cancer, RRT use, ventilator use, vasopressor use.

MBP, mean blood pressure; OASIS, Oxford Acute Severity of Illness Score; SOFA, Sequential Organ Failure Assessment; WBC, white blood cell; BUN, blood urea nitrogen; ALT, alanine aminotransferase; AST, aspartate aminotransferase; APAR, alkaline phosphatase to albumin ratio; RRT, renal replacement therapy.

**Figure 2:**
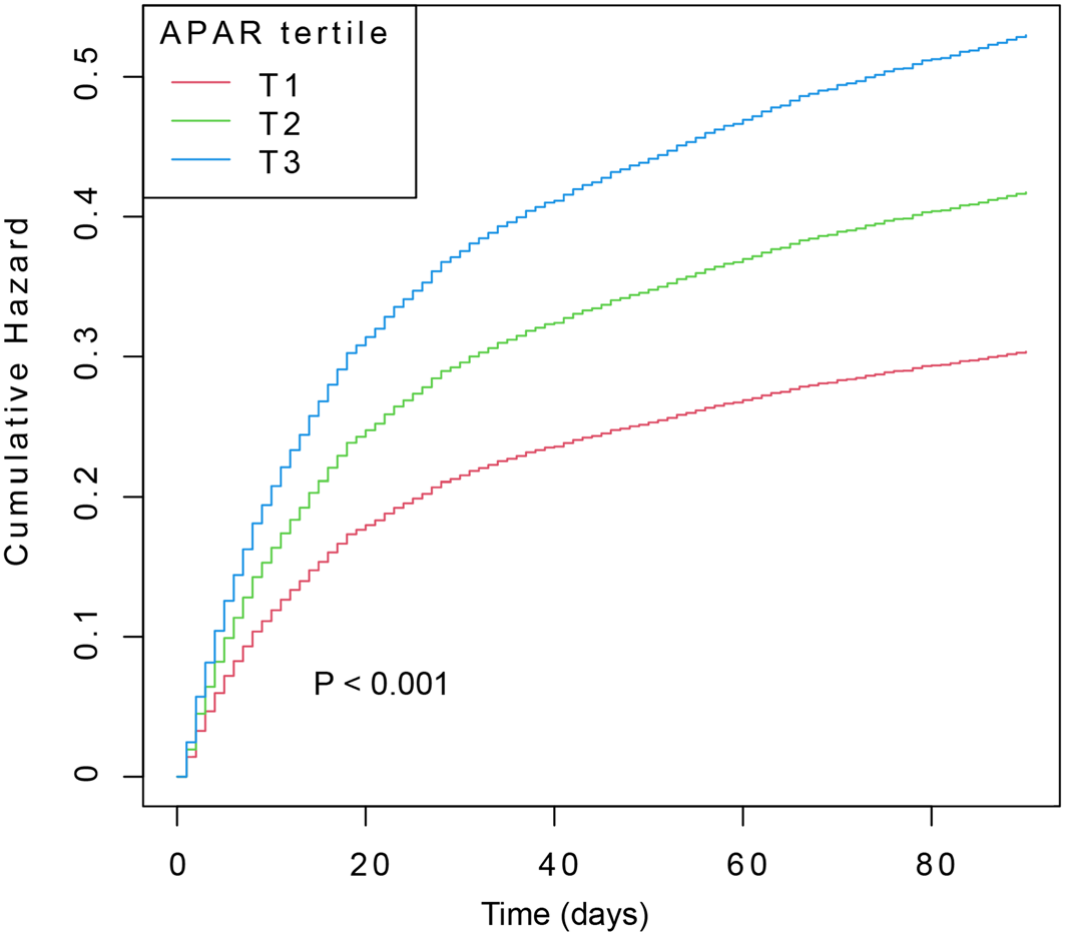
Kaplan–Meier analysis for 90-days survival probability in patients with sepsis. Groups based on tertile of APAR level (T1, APAR ≤ 2.2; T2, 2.2 < APAR ≤ 3.8; T3, APAR > 3.8). APAR, alkaline phosphatase to albumin ratio.

In order to evaluate the cumulative hazard at diverse APAR levels, we also created 90-days survival curves for sepsis stratified by APAR tertile (Fig. 2). The highest APAR tertile (T3) had the highest risk of 90-days mortality, which increased as baseline APAR increased (*P* value of the log-rank test < 0.001).

### Subgroup analysis

**Figure 3:**
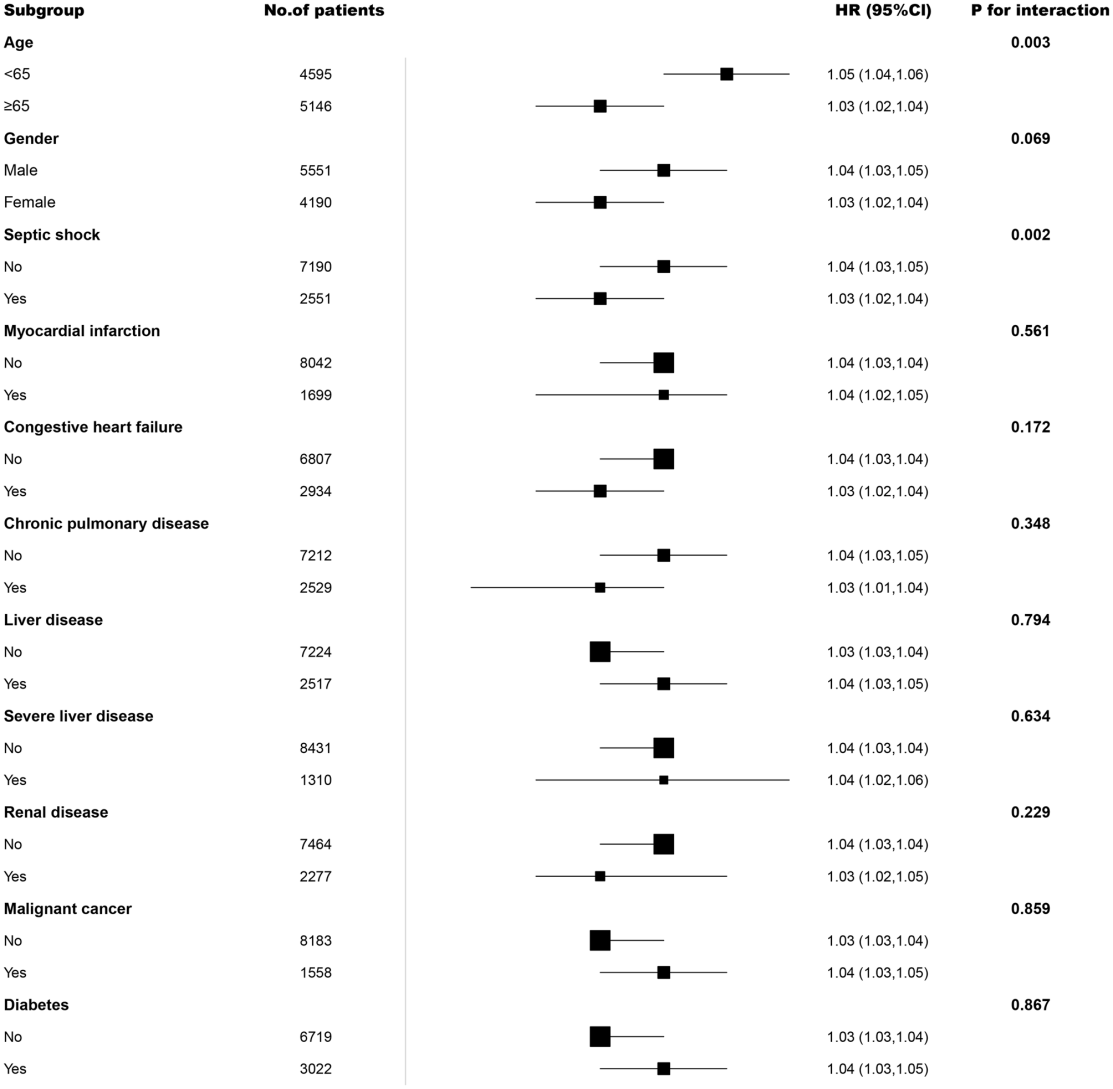
Subgroup analysis of the associations between APAR and 90-days mortality. HR (95% CI) were derived from Cox regression models. For age stratification, covariates were modified as in model 3 (Table 2); As for the other subgroups, they were adjusted for all confounders in model 3 (Table 2) except for the stratification factor itself. HR, hazard ratio; CI, confidence interval; APAR, alkaline phosphatase to albumin ratio.

In subgroup analyzes, we used age, gender, septic shock status, and comorbidity as stratification variables (Fig. 3). For age stratification, covariates were modified as in model 3. As for the other subgroups, they were adjusted for all confounders in model 3 except for the stratification factor itself. There was an age interaction between APAR and 90-days mortality (*P* for interaction = 0.003). With increasing APAR, 90-days mortality was significantly increased in the non-elderly subgroup (age < 65 years), and the HRs (95% CI) was 1.05 (1.04–1.06). There was also an interaction between APAR and 90-days mortality in septic shock (*P* for interaction = 0.002). In the other subgroupings, no significant interactions were identified (*P* for interaction > 0.05).

### ROC curves analysis

**Figure 4:**
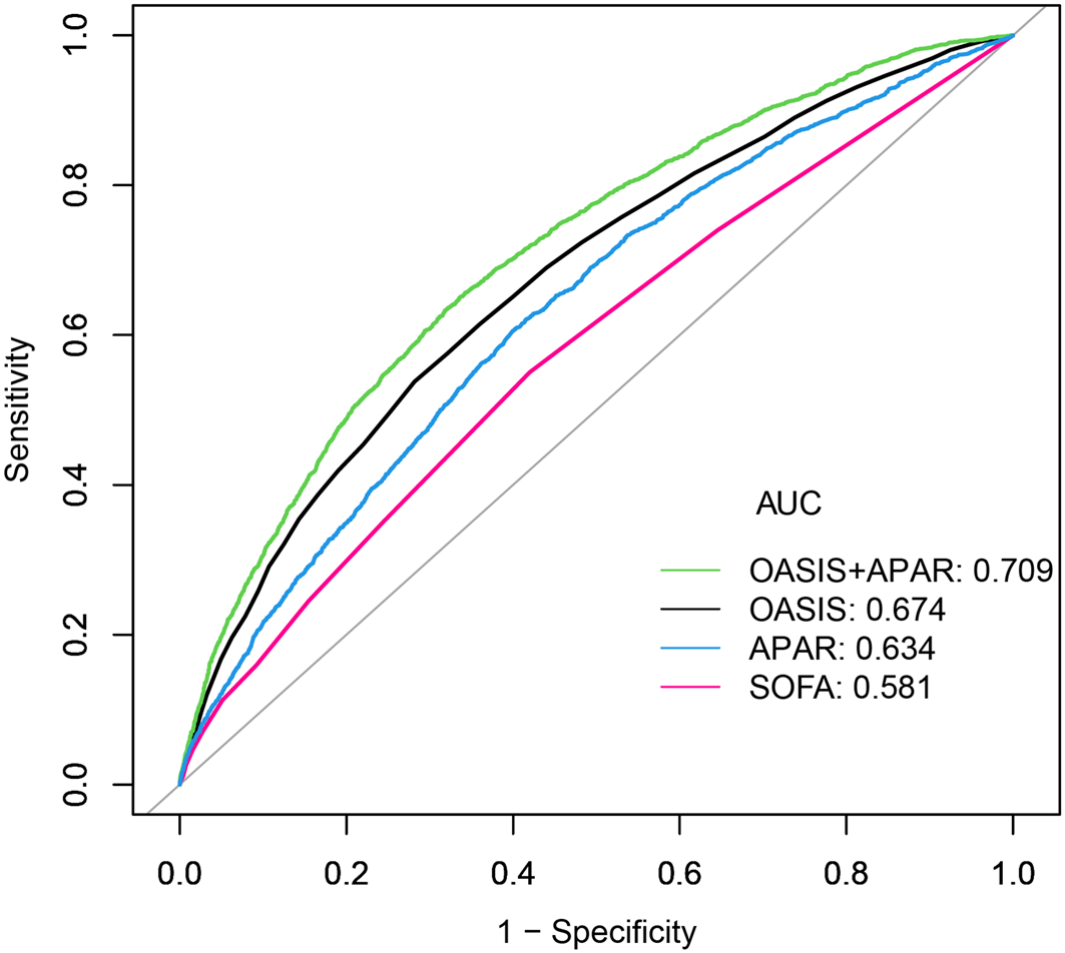
ROC analysis for the prediction of 90-days mortality. AUC, area under the curve; OASIS, Oxford Acute Severity of Illness Score; SOFA, Sequential Organ Failure Assessment; APAR, alkaline phosphatase to albumin ratio.

We further evaluated the prognostic power of APAR in sepsis using ROC curve analysis (Fig. 4, Table 3). The AUC of 90-days mortality for APAR, SOFA score, and OASIS was 0.634, 0.581, and 0.674, respectively. The specificity and sensitivity of SOFA were 58.0% and 55.0%, respectively. And the specificity and sensitivity of APAR were 60.0% and 60.6%, respectively. This suggests that APAR is a better predictor compared to SOFA. When APAR was combined with OASIS, an AUC of 0.709 was achieved. Compared to OASIS alone, the predictive power was significantly greater.

**Table 3:** AUC of APAR and other scoring systems for 90-days mortality.

	AUC	95% CI	Specificity	Sensitivity
SOFA	0.581	0.569–0.593	0.580	0.550
APAR	0.634	0.622–.6646	0.600	0.606
OASIS	0.675	0.663–0.686	0.718	0.538
OASIS + APAR	0.709	0.698–0.720	0.678	0.637

SOFA, Sequential Organ Failure Assessment; APAR, alkaline phosphatase to albumin ratio; OASIS, Oxford Acute Severity of Illness Score.

### Sensitivity analysis

We conducted three sensitivity analyzes. In both repeated analyzes using 1-year mortality (Supplementary Table 2) and analyzes after participants with missing values were removed (Supplementary Table 3), the correlation between APAR and clinical outcomes in sepsis patients was robust. In addition, by removing individuals with documented serum albumin infusion 48 h before the test of albumin and repeating the sensitivity analysis, the results remained stable (Supplementary Table 4).

## Discussion

In the current investigation, we found an independent association between APAR and sepsis prognosis. Elevated APAR levels were significantly associated with higher mortality. This is the first investigation to examine the connection between APAR and sepsis prognosis in our knowledge.

It is known that in response to infection, Toll-like receptors (TLR) bind to bacterial-derived lipopolysaccharide (LPS) and trigger an inflammatory response by activating the nuclear factor-kB (NF-kB) signaling pathway, which leads to the secretion of inflammatory cytokines, both pro-inflammatory and anti-inflammatory, further boosting hematopoiesis, phagocytosis, and leukocyte recruitment^24^. However, ALP can reduce the release of pro-inflammatory cytokines via the LPS-TLR4 pathway^25^. Previous studies have shown that ALP levels are positively correlated with CRP and neutrophil-to-lymphocyte ratio^26,27^. Elevated ALP levels may be caused by infection, or this higher anti-inflammatory effect of ALP may lead to unfavorable host defense^28,29^. Recent research suggests that higher ALP levels are linked to higher infection-related mortality and that ALP may be an inflammation biomarker^11,28^. Serum albumin has the ability to reflect inflammation and nutritional status. Albumin also has a role in regulating osmolality, as well as inhibiting the activation and aggregation of platelets^30–32^. In addition, albumin can alter the pharmacokinetics of antimicrobial drugs^33,34^. Albumin has been recognized as a biomarker for critical illness, and hypoalbuminemia in sepsis is related to a bad prognosis^35^.

APAR is a new indicator for binding albumin and alkaline phosphatase. Recent studies have found that this ratio is a reliable predictor of malignancy prognosis^36,37^. A similar result was also obtained in non-alcoholic fatty liver disease^38^. In addition, APAR is a good predictor in coronary artery disease patients^13^. And high APAR is related to the outcomes of acute kidney injury^14^. However, no research has indicated a relationship between APAR and sepsis.

Therefore, 9741 patients were enrolled in this research to explore the correlation between APAR and prognosis, and the findings indicated that an elevated APAR was associated with higher 90-days mortality. It is essential to explain that the main sites of infection were the bloodstream and genitourinary systems, which may be because ICU patients are very sick and suffer from multiple infections. Blood and urine are relatively easy specimens to obtain. Moreover, this significant correlation persisted as a categorical or continuous variable after adjustment. Higher APAR levels were linked to increased 90-days mortality in sepsis, according to a Kaplan–Meier analysis of the cumulative risk. In subgroup analysis further, there is an interaction between APAR and age. A possible reason for this is that older adults (≥ 65 years) have a weaker inflammatory response^39^. Elderly patients with sepsis impact clinical outcomes due to age-related physical and functional deterioration, and non-older adults are more sensitive to variations in APAR. There was also an interaction between septic shock and APAR with 90-days mortality. The correlation between APAR and 90-days death remained stable across subgroups. It is worth noting that albumin and alkaline phosphatase are commonly used in clinical practice to evaluate liver function. Among them, the ALB level is an indicator of the protein synthesizing capacity of the liver. And ALP is mainly concentrated in the liver. Therefore, when liver injury occurs, circulating ALP levels are elevated^40^. However, in our subgroup analysis, the results of this correlation remained robust in the population with liver disease and severe liver disease. To further validate the stability of APAR in relation to sepsis prognosis. We performed a sensitivity analysis of 1-year mortality in addition to the analysis of 90-days mortality. The results showed that APAR was associated with 1-year death in sepsis, and the correlation was somewhat stable.

ROC curve analysis showed that APAR has good predictive value in 90-days death in sepsis patients. We observed that the AUC of APAR was superior to that of SOFA. The AUC increased significantly with APAR combined with OASIS. Moreover, the 95% CI of AUC in Table 3 indicated that the AUC of APAR combined with OASIS was statistically different from that of OASIS. APAR combined with OASIS further improved the predictive ability of OASIS for sepsis death. The outcome indicates that APAR might be a more valuable indicator to enhance the ability of clinicians to obtain prognoses for patients with non-septic shock. It is worth discussing that the AUC of SOFA in the present study was 0.581. The AUC of SOFA in the present study was slightly lower compared to some studies^14,19^. We have referred to some literature, and we speculate that the difference may be due to the difference in the population selection, and the selection of our study population was septic patients in the ICU of the MIMIC database. Moreover, through the literature, we can know that the prognostic value of SOFA for in-hospital death and 28/30 days death is different^41^. SOFA has more excellent prognostic value in assessing in-hospital death compared to 28/30-days death. This may indicate that APAR can have a more excellent prognostic value in the medium and long term and has greater clinical significance. The population with missing APAR was excluded from our study, which may have also affected our results. In the Han et al*.* study, we can also see that SOFA has a similar prognostic value^42^. Although the difference in AUC between APAR and OASIS is small, dynamic monitoring of OASIS is difficult and costly due to the large number of markers involved, which is burdensome for both patients and physicians. APAR involves fewer markers, and rapid, low-cost test kits have been developed for detecting APAR, which significantly reduces the time, cost, and money spent on monitoring. This dramatically reduces the time and monetary costs of monitoring.

Patient status at the time of admission is vital for prognostic assessment, and it is significant to perform admission assessment in critically ill patients. Therefore, the timing of the APAR test is essential to help the physician. The variables included in this study were all tests performed within 24 h of admission. Therefore, we suggest that assessment within 24 h of admission will be more meaningful. In order to lower mortality in sepsis patients, this research may aid clinicians in identifying patient prognoses early.

### Strengths and limitations

Our research has several advantages. First, this is a study that used a sizable sample of real-world data. Second, we used rigorous statistical adjustments to reduce residual confounders. Third, by analyzing APAR as categorical and continuous variables, we enhanced the reliability of the findings. We also performed subgroup analyses to assess the association of sepsis with 90-days mortality in each subgroup to validate that the results were robust. Survival time was well documented in our study population, and therefore, survival time was adjusted in both regression analysis and survival analysis. In addition, we compared APAR with validated scores to better demonstrate the prognostic value of APAR. APAR is an objective indicator and easy to calculate, and more practical for clinical use.

However, our study also has some limitations. First, selection and confounding biases were challenging to avoid despite our best efforts to account for possible confounders and conduct subgroup analysis, which is an inherent drawback of retrospective investigations. Second, many laboratory test results for CRP, lactate, and PH were missing from our included population. Therefore, we did not adjust for these variables when performing regression analyses, which would have resulted in increased confounding. Third, we included a population of septic patients in the intensive care unit, which would affect the generalizability of this study. Moreover, we included only the first ICU admission in the analysis for patients with multiple ICU admissions, which may have created a selection bias. We selected only a single database, and the study population was not comprehensive enough. Due to the lack of external validation, this may result in our conclusions being limited. It may not be suitable for patients in other countries or regions. In addition, we primarily analyzed the 90-days mortality outcome, although the COX regression using survival time and the correlation analysis of 1-year mortality remained robust. However, there are limitations in the full spectrum of patient clinical outcomes. Finally, our findings suggest that APAR is connected to a bad clinical prognosis in sepsis. These results are experimental and serve to generate hypotheses. The next step we need to take is to conduct higher-quality prospective studies with external validation.

## Conclusion

This research revealed that APAR was independently associated with mortality in sepsis, including both 90-days and 1-year mortality. In addition, APAR is a reliable predictor of 90-days mortality. A higher APAR suggests a poorer prognosis. APAR can be used as a biomarker independently or in combination with other indicators to help clinicians determine the prognosis of sepsis earlier and more effectively.

### Supplementary Information

Supplementary Tables.

## Data Availability

All data in this research were obtained from the MIMIC-IV database. These data can be found below: https://physionet.org/content/mimiciv/2.0/.
