# Supplementary material for: Association between alkaline phosphatase to albumin ratio and mortality among patients with sepsis
Source: Sci Rep. 2024 Feb 7;14:3170. doi: 10.1038/s41598-024-53384-7 (PMC10850091; doi:10.1038/s41598-024-53384-7)
Supplement: Supplementary file 1 — Supplementary Tables. [file 41598_2024_53384_MOESM1_ESM.docx]

**Table S1:** **Results of univariate Cox regression analysis of 90-day mortality**

| **Variables** | **HR (95% CI)** | ***P*-value** |
| --- | --- | --- |
| Age | 1.02 (1.02~1.02) | <0.001 |
| Gender, female | 1.03 (0.96~1.10) | 0.440 |
| Ethnicity, white | 0.88 (0.82~0.94) | <0.001 |
| Weight | 0.99 (0.99~0.99) | <0.001 |
| MBP | 0.99 (0.99~0.99) | <0.001 |
| Heart rate | 1.00 (1.00~1.01) | <0.001 |
| Respiratory rate | 1.03 (1.02~1.03) | <0.001 |
| Temperature | 0.73 (0.70~0.76) | <0.001 |
| SpO_2_ | 0.97 (0.96~0.97) | <0.001 |
| OASIS | 1.06 (1.05~1.06) | <0.001 |
| SOFA score | 1.12 (1.11~1.14) | <0.001 |
| Charlson comorbidity index | 1.16 (1.15~1.18) | <0.001 |
| WBC | 1.02 (1.01~1.02) | <0.001 |
| Hemoglobin | 0.94 (0.92~0.95) | <0.001 |
| Platelet | 1.00 (1.00~1.00) | 0.012 |
| Glucose | 1.00 (1.00~1.00) | 0.821 |
| Anion gap | 1.04 (1.03~1.04) | <0.001 |
| Potassium | 1.16 (1.13~1.20) | <0.001 |
| Chloride | 0.99 (0.99~1.00) | <0.001 |
| Sodium | 1.00 (0.99~1.01) | 0.863 |
| Albumin | 0.95 (0.95~0.96) | <0.001 |
| Creatinine | 1.06 (1.04~1.08) | <0.001 |
| BUN | 1.01 (1.01~1.01) | <0.001 |
| ALT | 1.00 (1.00~1.00) | 0.071 |
| AST | 1.00 (1.00~1.00) | <0.001 |
| ALP | 1.00 (1.00~1.00) | <0.001 |
| APAR | 1.06 (1.05~1.06) | <0.001 |
| Septic shock | 1.91 (1.77~2.05) | <0.001 |
| Myocardial infarction | 1.21 (1.11~1.32) | <0.001 |
| Congestive heart failure | 1.30 (1.21~1.39) | <0.001 |
| Chronic pulmonary disease | 1.07 (0.99~1.16) | 0.096 |
| Liver disease | 1.24 (1.14~1.33) | <0.001 |
| Severe liver disease | 1.35 (1.23~1.48) | <0.001 |
| Diabetes | 0.99 (0.92~1.07) | 0.838 |
| Renal disease | 1.27 (1.17~1.37) | <0.001 |
| Malignant cancer | 1.89 (1.74~2.05) | <0.001 |
| Bloodstream infection | 1.0 (reference) | - |
| Genitourinary infection | 1.11 (1.01~1.22) | 0.032 |
| Respiratory infection | 1.38 (1.08~1.77) | 0.011 |
| Gastrointestinal infection | 1.61 (1.24~2.09) | <0.001 |
| Other | 0.78 (0.49~1.23) | 0.277 |
| Unknown | 0.73 (0.65~0.83) | <0.001 |
| RRT use | 1.56 (1.36~1.79) | <0.001 |
| Ventilator use | 1.37 (1.24~1.52) | <0.001 |
| Vasopressor use | 1.58 (1.47~1.69) | <0.001 |

**Abbreviations:** MBP, mean blood pressure; OASIS, Oxford Acute Severity of Illness Score; SOFA, Sequential Organ Failure Assessment; WBC, white blood cell; BUN, blood urea nitrogen; ALT, alanine aminotransferase; AST, aspartate aminotransferase; ALP, alkaline phosphatase; APAR, alkaline phosphatase to albumin ratio; RRT, renal replacement therapy; LOS, length of stay;

**Table S2: Multivariable Cox regression analysis to assess the association between APAR and 1-year mortality**

| **Variables** | **Unadjusted model** | |  | **Model I** | |  | **Model II** | |  | **Model III** | |
| --- | --- | --- | --- | --- | --- | --- | --- | --- | --- | --- | --- |
|  | **HR (95% CI)** | ***P*-value** |  | **HR (95% CI)** | ***P*-value** |  | **HR (95% CI)** | ***P*-value** |  | **HR (95% CI)** | ***P*-value** |
| APAR | 1.06 (1.05~1.06) | <0.001 |  | 1.06 (1.05~1.06) | <0.001 |  | 1.05 (1.04~1.05) | <0.001 |  | 1.04 (1.03~1.04) | <0.001 |
| Tertile |  |  |  |  |  |  |  |  |  |  |  |
| T1 (APAR≤2.2) | Ref |  |  | Ref |  |  | Ref |  |  | Ref |  |
| T2 (2.2<APAR≤3.8) | 1.65 (1.51~1.79) | <0.001 |  | 1.62 (1.49~1.76) | <0.001 |  | 1.45 (1.33~1.57) | <0.001 |  | 1.40 (1.29~1.53) | <0.001 |
| T3 (APAR>3.8) | 2.36 (2.18~2.56) | <0.001 |  | 2.35 (2.17~2.54) | <0.001 |  | 1.99 (1.84~2.16) | <0.001 |  | 1.78 (1.64~1.94) | <0.001 |
| *P* for trend |  | <0.001 |  |  | <0.001 |  |  | <0.001 |  |  | <0.001 |

**Notes:** Model I adjusted for age, gender; Model II adjusted for model I plus OASIS, SOFA score, Charlson comorbidity index; Model III adjusted for Model II plus ethnicity, weight, MBP, heart rate, respiratory rate, temperature, SpO2, WBC, hemoglobin, platelet, anion gap, potassium, chloride, creatinine, BUN, ALT, AST, myocardial infarction, congestive heart failure, chronic pulmonary disease, liver disease, severe liver disease, renal disease, malignant cancer, RRT use, ventilator use, vasopressor use.

**Abbreviations:** MBP, mean blood pressure; OASIS, Oxford Acute Severity of Illness Score; SOFA, Sequential Organ Failure Assessment; WBC, white blood cell; BUN, blood urea nitrogen; ALT, alanine aminotransferase; AST, aspartate aminotransferase; APAR, alkaline phosphatase to albumin ratio; RRT, renal replacement therapy.

**Table S3: Sensitivity analysis after excluded patients with missing data**

| **Variables** | **Unadjusted model** | |  | **Model I** | |  | **Model II** | |  | **Model III** | |
| --- | --- | --- | --- | --- | --- | --- | --- | --- | --- | --- | --- |
|  | **HR (95% CI)** | ***P*-value** |  | **HR (95% CI)** | ***P*-value** |  | **HR (95% CI)** | ***P*-value** |  | **HR (95% CI)** | ***P*-value** |
| APAR | 1.06 (1.05~1.06) | <0.001 |  | 1.06 (1.05~1.06) | <0.001 |  | 1.05 (1.04~1.05) | <0.001 |  | 1.04 (1.03~1.04) | <0.001 |
| Tertile |  |  |  |  |  |  |  |  |  |  |  |
| T1 (APAR≤2.2) | Ref |  |  | Ref |  |  | Ref |  |  | Ref |  |
| T2 (2.2<APAR≤3.8) | 1.63 (1.47~1.80) | <0.001 |  | 1.60 (1.45~1.77) | <0.001 |  | 1.42 (1.28~1.56) | <0.001 |  | 1.35 (1.22~1.49) | <0.001 |
| T3 (APAR>3.8) | 2.36 (2.15~2.59) | <0.001 |  | 2.34 (2.13~2.57) | <0.001 |  | 1.95 (1.78~2.15) | <0.001 |  | 1.68 (1.52~1.86) | <0.001 |
| *P* for trend |  | <0.001 |  |  | <0.001 |  |  | <0.001 |  |  | <0.001 |

**Notes:** Model I adjusted for age, gender; Model II adjusted for model I plus OASIS, SOFA score, Charlson comorbidity index; Model III adjusted for Model II plus ethnicity, weight, MBP, heart rate, respiratory rate, temperature, SpO2, WBC, hemoglobin, platelet, anion gap, potassium, chloride, creatinine, BUN, ALT, AST, myocardial infarction, congestive heart failure, chronic pulmonary disease, liver disease, severe liver disease, renal disease, malignant cancer, RRT use, ventilator use, vasopressor use.

**Abbreviations:** MBP, mean blood pressure; OASIS, Oxford Acute Severity of Illness Score; SOFA, Sequential Organ Failure Assessment; WBC, white blood cell; BUN, blood urea nitrogen; ALT, alanine aminotransferase; AST, aspartate aminotransferase; APAR, alkaline phosphatase to albumin ratio; RRT, renal replacement therapy.

**Table S4: Sensitivity analysis after excluded patients who had received human serum albumin infusion 48 hours before albumin testing**

| **Variables** | **Unadjusted model** | |  | **Model I** | |  | **Model II** | |  | **Model III** | |
| --- | --- | --- | --- | --- | --- | --- | --- | --- | --- | --- | --- |
|  | **HR (95% CI)** | ***P*-value** |  | **HR (95% CI)** | ***P*-value** |  | **HR (95% CI)** | ***P*-value** |  | **HR (95% CI)** | ***P*-value** |
| APAR | 1.06 (1.05~1.06) | <0.001 |  | 1.06 (1.05~1.06) | <0.001 |  | 1.05 (1.04~1.05) | <0.001 |  | 1.04 (1.03~1.04) | <0.001 |
| Tertile |  |  |  |  |  |  |  |  |  |  |  |
| T1 (APAR≤2.2) | Ref |  |  | Ref |  |  | Ref |  |  | Ref |  |
| T2 (2.2<APAR≤3.8) | 1.65 (1.49~1.81) | <0.001 |  | 1.61 (1.46~1.78) | <0.001 |  | 1.44 (1.31~1.59) | <0.001 |  | 1.38 (1.25~1.52) | <0.001 |
| T3 (APAR>3.8) | 2.38 (2.17~2.61) | <0.001 |  | 2.35 (2.15~2.58) | <0.001 |  | 1.99 (1.81~2.18) | <0.001 |  | 1.72 (1.56~1.90) | <0.001 |
| *P* for trend |  | <0.001 |  |  | <0.001 |  |  | <0.001 |  |  | <0.001 |

**Notes:** Model I adjusted for age, gender; Model II adjusted for model I plus OASIS, SOFA score, Charlson comorbidity index; Model III adjusted for Model II plus ethnicity, weight, MBP, heart rate, respiratory rate, temperature, SpO2, WBC, hemoglobin, platelet, anion gap, potassium, chloride, creatinine, BUN, ALT, AST, myocardial infarction, congestive heart failure, chronic pulmonary disease, liver disease, severe liver disease, renal disease, malignant cancer, RRT use, ventilator use, vasopressor use.

**Abbreviations:** MBP, mean blood pressure; OASIS, Oxford Acute Severity of Illness Score; SOFA, Sequential Organ Failure Assessment; WBC, white blood cell; BUN, blood urea nitrogen; ALT, alanine aminotransferase; AST, aspartate aminotransferase; APAR, alkaline phosphatase to albumin ratio; RRT, renal replacement therapy.
